# Deep reinforcement learning empowers automated inverse design and optimization of photonic crystals for nanoscale laser cavities

**DOI:** 10.1515/nanoph-2022-0692

**Published:** 2023-01-12

**Authors:** Renjie Li, Ceyao Zhang, Wentao Xie, Yuanhao Gong, Feilong Ding, Hui Dai, Zihan Chen, Feng Yin, Zhaoyu Zhang

**Affiliations:** Shenzhen Key Laboratory of Semiconductor Lasers, School of Science and Engineering, The Chinese University of Hong Kong, Shenzhen, Guangdong, China,; Shenzhen Research Institute of Big Data (SRIBD), Shenzhen, Guangdong, China; Future Network of Intelligence Institute (FNii), Shenzhen, Guangdong, China; School of Science and Engineering, The Chinese University of Hong Kong, Shenzhen, Guangdong, China; Peng Cheng Laboratory, Shenzhen, Guangdong, China

**Keywords:** deep learning, inverse design, nanobeams, nanolasers, photonic crystals, reinforcement learning

## Abstract

Photonics inverse design relies on human experts to search for a design topology that satisfies certain optical specifications with their experience and intuitions, which is relatively labor-intensive, slow, and sub-optimal. Machine learning has emerged as a powerful tool to automate this inverse design process. However, supervised or semi-supervised deep learning is unsuitable for this task due to: (1) a severe shortage of available training data due to the high computational complexity of physics-based simulations along with a lack of open-source datasets and/or the need for a pre-trained neural network model; (2) the issue of one-to-many mapping or non-unique solutions; and (3) the inability to perform optimization of the photonic structure beyond inverse designing. Reinforcement Learning (RL) has the potential to overcome the above three challenges. Here, we propose Learning to Design Optical-Resonators (L2DO) to leverage RL that learns to autonomously inverse design nanophotonic laser cavities without any prior knowledge while retrieving unique design solutions. L2DO incorporates two different algorithms – Deep Q-learning and Proximal Policy Optimization. We evaluate L2DO on two laser cavities: a long photonic crystal (PC) nanobeam and a PC nanobeam with an L3 cavity, both popular structures for semiconductor lasers. Trained for less than 152 hours on limited hardware resources, L2DO has improved state-of-the-art results in the literature by over 2 orders of magnitude and obtained 10 times better performance than a human expert working the same task for over a month. L2DO first learned to meet the required maxima of *Q*-factors (>50 million) and then proceeded to optimize some additional good-to-have features (e.g., resonance frequency, modal volume). Compared with iterative human designs and inverse design via supervised learning, L2DO can achieve over two orders of magnitude higher sample-efficiency without suffering from the three issues above. This work confirms the potential of deep RL algorithms to surpass human designs and marks a solid step towards a fully automated AI framework for photonics inverse design.

## 1 Introduction

Inverse design of optical resonators [1, 2] is a crucial step in designing state-of-the-art nanoscale laser cavities [3] that realize classic photonic crystal lasers [4–6] finding broad applications in photonic integrated circuits, optical interconnects, and telecommunications. The inverse problem herein learns the hidden relationship between optical response and physical structure and typically involves retrieving a design topology for desired optical responses. Due to its seemingly “counter-intuitive” nature and a non-convex [7, 8] solution space, compared to the forward prediction problem, inverse design has been a major challenge [9] in the photonics community and been intensively studied in a multitude of disciplines [10–15].

**Figure 1:**
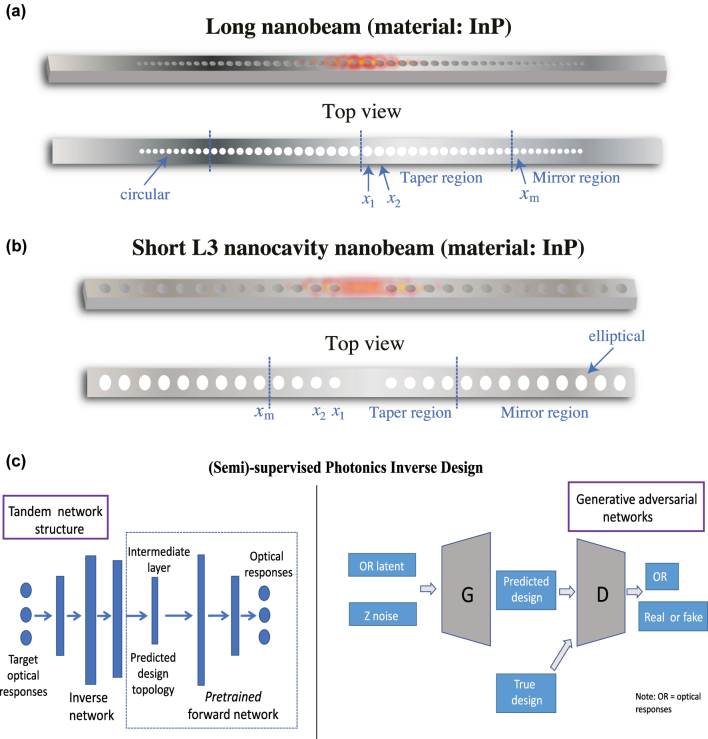
PC nanobeam laser cavities used in this work for inverse design and optimization via deep reinforcement learning and an overview of existing deep learning-based inverse design models. (a) 3D visualization of a typical long PC nanobeam and its top view. (b) 3D visualization of a typical short L3 nanocavity nanobeam and its top view. Indium Phosphide (InP) is chosen as the semiconductor laser material (i.e., the gain material). Physical configurations and optical characteristics of both nanobeams are shown in Table 1. Both nanobeams possess perfect symmetry both laterally and longitudinally. *x*_*i*_ is the *x*-coordinate of the *i*th air hole counting from the inside in the taper region and *x*_*m*_ is the *x*-coordinate of the holes in the mirror region. (c) Existing (semi)-supervised models for photonics inverse design, which takes target optical responses as input and outputs retrieved design parameters. Left: a tandem neural network structure built with MLPs, where the boxed region represents the pre-trained forward network. Right: a GAN-based model that is able to distinguish predicted design parameters from ground truths. This method is unable to perform optimization of the photonic structure. G and D in the figure denote the generator and discriminator, respectively. This work will address the flaws inherent in these DL models shown here. A detailed comparison between the method proposed in this work and existing DL methods can be found in the Supporting Information.

Since the 90s, many approaches to photonics inverse design have been proposed, mainly including: first-principle methods [16, 17], finite element analysis (FEA) or finite difference time domain (FDTD)-based simulation solvers [18, 19], and evolutionary [20, 21] or gradient-based [22, 23] optimization algorithms. While these approaches have historically yielded satisfactory results, they tend to demand heavy human involvement due to an iterative or trial-and-error nature and thus have defied automation. With the advancement of deep learning since circa 2012 [24, 25], researchers found new hope in the intelligent inverse design and optimization of nanophotonics [2, 9]. However, existing supervised and semi-supervised learning methods (Figure 1(c)) ultimately suffer from three unresolved problems: (1) the need for a large pre-collected dataset for training and/or the need for a pre-trained Multi-layer Perceptrons (MLP) network in tandem network architectures; (2) the undesired one-to-many mapping that leads to non-unique design solutions for a given optical response; and (3) the inability to perform optimization of the photonic structure beyond inverse designing; in other words, the inverse designed structure simply mimics or replicates the initial design it started with and the optical features of the structure do not receive any improvement or optimization. Among the above, point 1 is the toughest to overcome primarily because deep learning is known to be highly data-hungry. Also, physics-based simulators solving Maxwell’s equations are generally time-consuming, thus generating large-scale datasets can become inefficient or even infeasible. On top of that, most nanophotonic devices are proprietary within individual research groups, making it difficult to release/access open-source datasets for the community. Points 2 and 3 pose a major challenge to the photonics community as well. Although a plethora of existing works (see Figure 1(c)) have attempted to use tandem MLP networks [26, 27], Generative Adversarial Networks (GANs) [28, 29], Variational Autoencoder (VAE) [30, 31], and iterative optimization with DNNs [32] to inverse design various photonic devices, these prior art have been limited by the above unresolved issues.

Reinforcement Learning (RL) started out as a purely mathematical model over half a century ago [33] and in recent years has been successfully applied to solving increasingly more real-world tasks [34–45] with profound scientific and engineering significance. Google DeepMind’s AlphaGo Zero [46] and AlphaStar [47] are prominent hallmarks of RL’s ability to reach or even exceed the abilities of human beings. While conventional (semi)-supervised learning with deep neural networks (DNNs) has recently been applied in photonics forward [48–50] and particularly inverse designs [26], [27], [28], [29, 32, 51, 52], RL is still a relatively under-explored realm to date. Nonetheless, since RL requires NO pre-collected training data (and instead explores on the fly), is free of the non-uniqueness issue, and can provide optimizations of the photonic structure beyond inverse designing, it has a clear edge in the inverse problem. For example, recently, Sajedian et al. adopted deep RL to optimize dielectric nanostructures and high-transmission color filters [45, 53] in two separate works, marking the inception of RL in photonics design. More recently, Sui et al. inverse designed digital nano-materials using deep RL and demonstrated a robust convergence of the model [54]. In 2021, Mirhoseini et al. posed chip floorplanning as a deep RL problem to design the newest of Google’s tensor processing unit accelerators that surpassed strongest baseline models [38]. In 2022, Kuprikov et al. demonstrated a deep RL approach that was successfully used for the control of the generation of dissipative solitons in mode-locked fiber laser system [44]. However, there’s no existing literature that inverse design optical resonators or laser cavities using RL algorithms.

RL algorithms are best suited for solving complex high-dimensional (and possibly non-convex) optimization problems [55], especially when there’s a large design parameter space to explore. In our case, even one variable (e.g., picking the number of air holes in the nanobeam) has an enormous state – action space and degree of freedom. Thus, given the scale and difficulty of the photonics inverse problem, we developed a deep RL method that, through repeated episodes (sequences of states, actions and rewards), manages to explore a sizeable design parameter space looking for the optimal solution. We named the proposed method *Learning to Design Optical-Resonators* (*L2DO*). Because of the existence of a wide variety of RL algorithms, we opted to experiment with Deep Q-learning (DQN) [39] by Google DeepMind and Proximal Policy Optimization (PPO) [56] by OpenAI for L2DO’s implementation. To demonstrate the power of L2DO, two different photonic crystal (PC) nanobeam cavities [57, 58] are investigated in this work. In our experiments we show that, with a greater volume and variety of data samples accumulated during training, L2DO learns to be both faster and more stable at generating unique design parameters that satisfy given optical responses, thus solving the inverse design problem. In addition, L2DO can optimize good-to-have optical quantities that are also important for an ideal laser cavity. Finally, using several novel modifications to the model and training process, the sample efficiency (i.e., exploration rate) and convergence speed of L2DO were successfully improved by two and a half times, which helped alleviate the expensiveness of training the RL model. L2DO brings us closer to a future in which nanophotonic researchers are assisted by autonomous artificial agents with potentially vast optimization experience and superhuman efficiency. To the best of our knowledge, this work is the first to leverage deep RL for inverse designing and optimizing nanoscale laser cavities with a diversified array of tunable parameters.

## 2 Methods and results

**Objective overview.** To demonstrate L2DO’s effectiveness and efficiency in inverse designing different laser cavities, we chose to investigate a long PC nanobeam and a short L3 nanocavity nanobeam as schematically visualized in Figure 1(a) and (b). For simplicity, the former will be denoted as “long nanobeam” and the latter “L3 nanobeam” going forward in this text. Each nanobeam is divided into a Taper region and a Mirror region, respectively, according to their unique air hole size distributions. The *x*’s refer to the *x*-coordinate of each air hole, where *x*_1_, *x*_2_, … are the *x*-coordinate of the *i*th air hole counting from the inside in the Taper region and *x*_*m*_ is the *x*-coordinate of the holes in the Mirror region. Both nanobeams have Indium Phosphide (InP) as the gain (i.e., active) material. Nanobeam cavities are commonly used as laser sources in photonic integrated circuits (PICs), interconnects, and telecommunications when coupled to waveguides and optic fibers.

Both nanobeams’ initial specifications are summarized in Table 1, including their respective design parameters and optical responses. Design parameters are set with discretion according to the wafer specifications, fabrication capabilities, and special lasing requirements. In addition, both nanobeams are designed to be perfectly symmetric (i.e., left and right sides are mirror image of each other). Among others, a key difference is that air holes in the L3 nanobeam are elliptical, whereas those in the long nanobeam are circular. This design choice was made on a case-by-case basis with a special focus on maximizing the *Q* factor of each nanobeam. Finally, *Q* factors, resonance (operational) wavelengths *λ*, and modal volumes *V* are calculated by 3D FDTD simulations. Due to its more complex structure (i.e., hole shape, thickness etc.), the L3 nanobeam’s FDTD simulations take more time to finish compared to the long nanobeam, as shown in Table 1. The simulation time will play a major role in our subsequent design of the deep RL algorithm.

**Table 1:** Initial design parameters and optical responses of the long nanobeam (top) and L3 nanobeam (bottom), respectively. Initial design parameters are determined according to wafer dimensions, fabrication limitations, and specific application needs. Optical responses are calculated by 3D FDTD simulations using the initial design parameters. “Sim time” denotes how much time it takes to simulate the nanobeam once. Since the L3 nanobeam has elliptical holes, its radius is in fact semi-minor (major) axes.

Design params	Values	Optical responses	Values
**Long nanobeam**			
Length	20 μm	*Q*	3e+6
Width	700 nm	*V*	0.686 ${\left(\frac{\lambda }{n}\right)}^{3}$
Thickness	220 nm	*λ*	1550 nm
Material	InP	Sim time	2.5 min
No. of holes	25		
No. of taper holes	15		
No. of mirror holes	10		
Shape of holes	Circular		
Radius of holes	See Supporting Information		
Locations of holes	See Supporting Information		
**L3 nanobeam**			
Length	6.2 μm	*Q*	8e+4
Width	300 nm	*V*	1.079 ${\left(\frac{\lambda }{n}\right)}^{3}$
Thickness	500 nm	*λ*	950 nm
Material	InP	Sim time	4.5 min
No. of holes	13		
No. of taper holes	4		
No. of mirror holes	9		
Shape of holes	Elliptical		
Radius of holes	See Supporting Information		
Locations of holes	See Supporting Information		

**The inverse design problem defined.** With the initial design parameters and optical responses introduced, the inverse design task to be fulfilled by the proposed L2DO is formally defined as follows:

1. *Increase* the *Q* factor of the long nanobeam and L3 nanobeam to a target maximum of 5e+7 and 3e+6, respectively, from their initial values and *retrieve* the corresponding **unique** design parameters (REQUIRED, the higher the better);
2. *Maintain* the modal volume *V* of both nanobeams at or below their initial levels (good-to-have, the smaller the better);
3. *Constrain* the resonance wavelength *λ* of both nanobeams to their initial values ± 50 nm of tolerance (good-to-have, the closer the better),

where “REQUIRED” means L2DO must solve for a unique set of design parameters that satisfies given target optical responses (i.e., *Q* factors). The target maxima are chosen as 5e+7 and 3e+6 because experimentalists in our group determined those magnitudes to be ideal for high-quality lasing but hard to obtain with other inverse design approaches. “good-to-have”, on the other hand, represents some additional optical features L2DO needs to optimize. For example, keeping the modal volume small can enable tight on-chip integration and reduce device dimensions while constraining the shift of wavelengths is important for lasing with precise frequencies for applications in telecommunications.

**Deep RL algorithms and their elements.** To address the challenging photonics inverse design problem, we developed a deep RL method capable of seamlessly generalizing across structures with increasingly more experience and higher efficiency accumulated over time. In other words, L2DO learns from its own experiences to inverse design a variety of optical resonators and laser cavities. Specifically, the RL problem can be formulated as a sequential Markov decision process (MDP) [33], because this allows us to more easily incorporate the inverse problem’s core attributes. We list five key MDP elements used in our L2DO:

(1) *States* encode information about the structural (design) parameters, including the material’s refractive index, the spatial arrangement of components, the number, size and shape of each component, and the length, width, and thickness of the whole device, among others.
(2) *Actions* are all possible changes in the value of the above state encodings, without violating any hard constraints on size or locations. Optimal actions are predicted by the RL agent’s policy neural network and are expected to traverse a large portion of the range of states.
(3) *Policy* represents the key part of an RL agent that receives a state and makes decisions for what actions to take. A policy is generally parametrized by DNNs and can be considered as the “brain” of an RL model.
(4) *Environment* is the FEA/FDTD-based simulation software solving Maxwell’s equations and it takes in an action and outputs the next state and the associated reward. Simulating photonic components routinely takes several minutes or even hours. To alleviate the computational burden induced by simulations, a DNN could be introduced to approximate the environment; more details about this approximation will be given later in this section.
(5) *Rewards* are proportional to how close we get to the specified design targets, and good actions earn higher rewards. Design targets are defined by preset maxima (e.g., optical responses like the *Q*-factor, modal volume etc.) to reach, as well as other good-to-have features (e.g., resonance wavelength, bandgap width etc.) to optimize.

Full definitions of these elements are included in the Suppporting Information.

**Figure 2:**
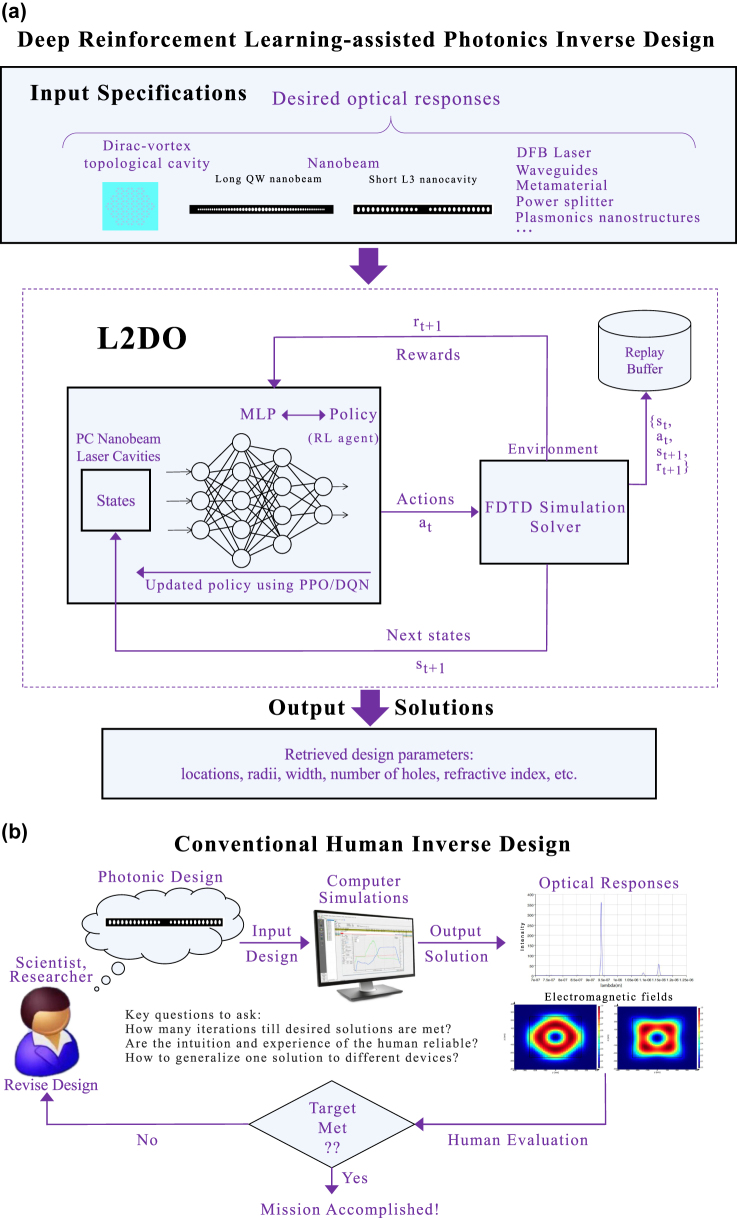
Comparison between deep RL-enabled (this work) and conventional human-centered inverse design methodologies. (a) Full details of the deep RL-based implementation of L2DO for photonics inverse design. L2DO takes in the desired optical specifications of certain photonic devices and delivers the solved design parameters. Both DQN and PPO algorithms are seamlessly incorporated within L2DO. The policy (RL agent) is implemented with a 4-layer MLP network whereas the environment is realized by an FDTD-based simulation solver (or optionally a DNN). Data sequences are stored in a replay buffer for data sample reuse and breaking sample correlations. (b) Human-centered iterative inverse design approach, which is still the most common practice in existence today. This conventional approach, relying on the intuition and experience of the human expert, typically takes over a month or even months to yield a satisfactory photonic design.

**Implementation of L2DO by deep RL.** Figure 2(a) schematically illustrates the full deep RL implementation of L2DO, containing the input and output, the policy network, the environment, actions, states, and rewards, etc. L2DO takes desired optical responses as input and yields the retrieved design parameters as output. Both DQN and PPO algorithms are completely integrated and realized with this model design. Here, state is also sometimes referred to as observation in the literature. The policy network, typically realized by DNNs, is taken to be an MLP neural network in our case. The policy represents the autonomous RL agent that decides what action to take during inverse designing and can be considered as the “brain” of an RL model. The goal of the policy is to gradually maximize the cumulative reward. One complete loop inside the box with dotted lines in Figure 2(a) constitutes one training step of L2DO, and multiple steps constitute a typical training episode in RL. In our case, we set the maximum number of steps within an episode (also known as the horizon) to be 250. Full model specs and hyperparameters of L2DO can be found in the extended data in the Supporting Information. Figure 2(b) pictorially illustrates the commonly practiced human-centered inverse design mythology, where a human expert (usually a scientist or researcher) devises a specific photonic design, enters it into a simulation solver, computes the output optical responses, evaluates the results by comparing them to desired targets, and revises the design if targets unsatisfied. This iterative approach, heavily relying on the knowledge and experience of the human expert, is highly labor-intensive, slow, and illy generalizable. We aspire to supersede this human-centered approach by our deep RL-empowered L2DO.

**Figure 3:**
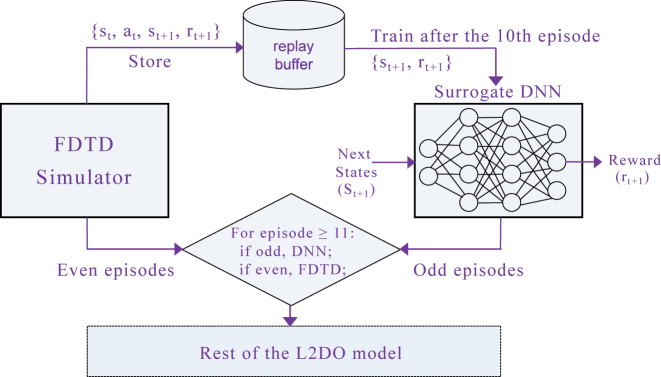
The novel DNN/Simulator Alternating technique (DSA), where the surrogate DNN and the actual FDTD simulator take turns to function as the environment of L2DO. Input to the DNN is next states and output is rewards. The DNN is only activated after the first *E* episodes, which is set to be 10 for illustrative purposes in this diagram. Right before the activation, the DNN is trained on data samples collected from FDTD during the first *E* episodes. After the activation, the DNN and the simulator alternate between odd and even episodes, respectively. Samples during the episodes when the simulator is used are still stored to the replay buffer for continuously training the DNN down the road. DSA is expected to cut down training time by a large portion.

Next, the expensiveness associated with training RL models is addressed. Although RL does not require any pre-collected training data, it still has to sample data and do explorations on the fly. This could cause issues like a low sample efficiency and difficult training convergence especially at the presence of a time-consuming (i.e., expensive) environment. In this work, we devised and propose a novel DNN/Simulator Alternating technique (DSA) (see Figure 3) that works in harmony with L2DO, where a surrogate DNN is introduced to approximate the FDTD simulator to reduce the computational cost of simulations and boost L2DO’s sample efficiency. This surrogate DNN essentially predicts rewards from given states. More details in the Supporting Information.

**Figure 4:**
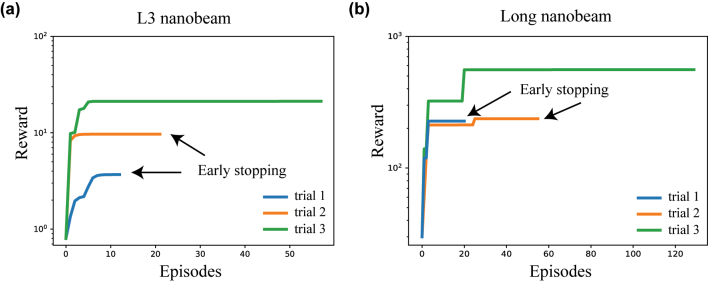
Learning curves of L2DO, plotted as rewards versus episodes. Vertical axes are in log-scale. (a) Convergence trends of the L3 nanobeam trained with L2DO. Trial 1 and 2 were terminated earlier because the reward stopped growing for over 50 consecutive steps. Trial 3 represents the optimal learning curve trained for 58 episodes. (b) Convergence trends of the long nanobeam trained with L2DO-DSA, where trial 3 represents the optimal learning curve trained for 131 episodes. Early stopping is utilized as well. Choice of DSA for either the L3 or the long nanobeam is explained in the Supporting Information.

**Learning curves of L2DO and the retrieved designs.** Training results of L2DO are included in this part where studies on both the long and L3 nanobeams are demonstrated. The RL code was fully written in Python strictly following the algorithmic model illustrated in Figures 2 and 3, and popular machine learning libraries like Pytorch, gym, and Ray RLlib were extensively utilized in our program. Figure 4 showcases the learning curve plots (rewards vs. episodes) where the vertical axes are plotted in log-scale. Figure 4(a) shows the training convergence of using L2DO to inverse design the L3 nanobeam. Three separate trials were done and trial 3 (green) represents the optimal solution for the inverse design problem. Trial 1 (blue) and trial 2 (orange)’s training were terminated earlier because their respective rewards showed no improvement for over 50 consecutive steps. Similarly, Figure 4(b) shows the three trials for inverse designing the long nanobeam by L2DO-DSA and the optimal trial is also colored in green. The rewards in Figure 4 are calculated by Equation 6. Hyperparameters of the optimal runs are included in the extended data in the Supporting Information.

**Figure 5:**
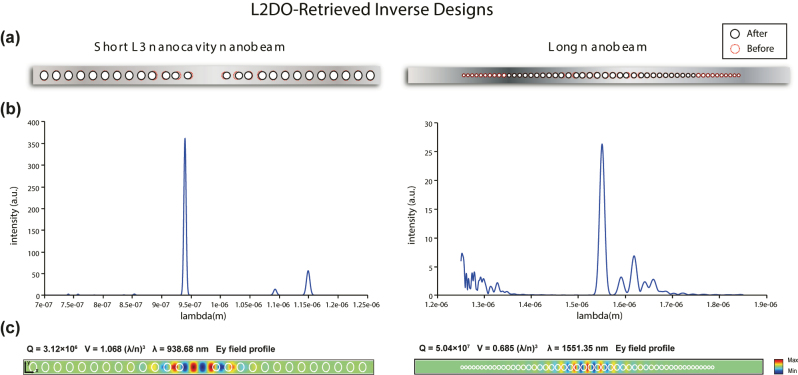
Physical structures, spectra, and electric field profiles (*E*_*y*_) of the inverse designed nanobeams from Table 2. Left panel: L3 nanobeam’s solved structure, spectrum and electric field profile, respectively; right panel: long nanobeam’s solved structure, spectrum and electric field profile, respectively. In (a), red dashed circles correspond to the original holes, whereas black solid circles represent the resulting holes from L2DO’s inverse design solution. Results in (b) and (c) are generated by 3D FDTD simulations. *y*-axes of the spectra in (b) represent the intensity of the EM field whereas *x*-axes represent wavelengths. A single highest peak in a spectrum corresponds to a single resonance mode. *E*_*y*_ field plots in (c) display the electromagnetic resonance modes of the retrieved nanobeam structures, where the verified *Q*, *V*, and *λ* are also labelled. Resonance modes are necessary for lasing operations.

Based on the optimal curves in Figure 4, a unique set of design parameters retrieved for both nanobeams that successfully met the target maxima are summarized in Table 2. The set of solved design parameters corresponds to the state space defined in Table 2 in the Supporting Information. The physical structures of the inverse designed nanobeams are schematically illustrated in Figure 5(a), where black circles and the red dashed circles correspond to the solved and initial holes, respectively. We then verified that desired optical responses were indeed satisfied by L2DO by feeding the solved design parameters into the FDTD simulator and examining the output optical responses, which are also listed in Table 2 (check-marked). It is clearly observed that the FDTD-verified values (i.e., *Q*, *V*, and *λ*) are highly aligned across the board with the target values (i.e., ground truths) marked with stars and small differences are present but well under the margin of error. More importantly, the verified *Q*-factors (3.12 × 10^6^ and 5.04 × 10^7^, respectively) not only solved the inverse design problem by substantially improving from the initial values (8 × 10^4^ and 3 × 10^6^, respectively, as shown in Table 1) but also even exceeded the target maxima (3 × 10^6^ and 5 × 10^7^) by an appreciable amount (the higher, the better). Moreover, the verified *V*’s are calculated to be smaller than the target values (the smaller, the better) while the verified *λ*’s are shifted away from the target values by differences well under the tolerance of ±50 nm (the closer the better). These results mean that L2DO not only satisfied the REQUIRED and good-to-have tasks simultaneously, but also provided us extra bonuses we did not originally ask for. The target optical responses (starred) are extracted from Table 1 and the definition of the inverse problem presented earlier, whereas the verified values using the solved design parameters are accompanied by check marks in Table 2.

**Table 2:** L2DO-retrieved unique set of design parameters for the L3 nanobeam (top) and long nanobeam (bottom), respectively, are shown in the first two columns. Target optical responses are marked with stars. Also tabulated are the FDTD-verified optical responses using the solved design parameters (check-marked) and human expert-reported values. The model, algorithm (algo), and total training time of each nanobeam are written in the Table, as well.

L3 nanobeam			
Model: L2DO	Algo: DQN	Training time: 88 hours	
Solved design params	Values (nm)	Target opt. resp.	Values
*x*_1_	2.85	Q*	3e+6
*x*_2_	4.7	V*	1.079 ${\left(\frac{\lambda }{n}\right)}^{3}$
*x*_3_	−0.45	λ*	950 nm
*x*_4_	2.0	**Verified opti. resp.**	**Values**
*a*_1_	1.3	*Q*	3.12e+6 ✓
*a*_2_	−2.8	*V*	1.068 ${\left(\frac{\lambda }{n}\right)}^{3}$ ✓
*x*_*m*_	0.0	*λ*	938.68 nm ✓
		**Human expert** **[59]:** *Q*	2.0e+5

Long nanobeam			
Model: L2DO-DSA	Algo: PPO	Training time: 152 hours	
Solved design params	Values (nm)	Target opt. resp.	Values
*x*_1_	−0.10	Q*	5e+7
*x*_2_	0.0	V*	0.686 ${\left(\frac{\lambda }{n}\right)}^{3}$
*x*_3_	0.10	λ*	1550 nm
*x*_4_	−0.85	**Verified opti. resp.**	**Values**
*x*_5_	0.10	*Q*	5.04e+7 ✓
*x*_6_	0.60	*V*	0.685 ${\left(\frac{\lambda }{n}\right)}^{3}$ ✓
*r*	−0.15	*λ*	1551.35 nm ✓
		**Human expert**: *Q*	1.3e+7

To better illustrate the advantage of L2DO, best *Q*-factors reported by a human expert [59] manually tuning the exact same nanobeams are tabulated in Tables 2 and 3 for comparison as a baseline. It is observed that the FDTD-verified *Q*’s definitively beat the human results by a large margin for both nanobeams (≈10 times and 5 times, respectively). *λ* and *V* reported by the human, on the other hand, are close to the L2DO-solved values in magnitude and thus are not listed (the desired *λ* and *V* are easier to achieve than *Q* because they do not require any major increase in magnitude (i.e., optimization)). Furthermore, human experts often need to spend over a month on the device optimization process as shown in Figure 2(b), whereas it only took us a few days to tune and train L2DO using hardware resources no more than a few workstations. Full specifications of the computing resources used are included in the Supporting Information. Finally, all solved design parameters fall within the bounds set by the state spaces in Table 2 in the Supporting Information, indicating a good tractability of the algorithm. Therefore we conclude that, without any specific expert knowledge or suffering from the one-to-many mapping issue, L2DO has indeed fulfilled the inverse design task defined before with above-expectation and superhuman performance.

**Table 3:** Comparison of results in this work to best baselines from the literature as well as a human expert. Only the *Q*-factor is used as the performance metric here. The long nanobeam is chosen as the representative because its number of holes is similar to the baseline models.

Source	*Q* factor	Material	No. of holes	Method	Time spent
L2DO (this work)	5.04e+7	InP	25	Deep RL	152 h
Kim et al. [60]	1e+5	InGaAsP	≈20	Manual	N/A
McCutcheon et al. [61]	1e+6	SiN_*x*_	24	Manual	N/A
Quan et al. [62]	1e+9	Si	40	Manual	N/A
Human expert [59]	1.3e+7	InP	25	Manual	1.5 months

We have also included best results from the literature [60–62] as baselines and have them tabulated along with our results for comparison in Table 3. Since the semiconductor materials/nanobeam dimensions used in existing literature are generally different from ours, their results might not be suitable for a direct comparison of metrics to ours; nonetheless, they can provide a perspective on how well our L2DO performed. For instance, Quan et al. [62] manually designed a nanobeam that reported a high *Q* of 1e+9, where the material is *Si* and the nanobeam has 40 holes on each side. McCutcheon et al. [61] manually designed a nanobeam that reported a high *Q* of 1e+6, where the material is SiN_*x*_ and the nanobeam has 24 holes on each side. Kim et al. [60] manually designed a nanobeam that reported a high *Q* of 1e+5, where the material is InGaAsP quantum wells and the nanobeam has around 20 holes on each side. In this work, we leveraged deep RL to design a long nanobeam reporting a high *Q* > 5e+7, where the material is InP and the nanobeam has 25 holes on each side. As we can see here, each work (including ours) used a different material (i.e., refractive index) and number of holes in their nanobeam, among other design choices, which resulted in varying *Q* factors. Compared to Kim et al. [60] which adopted a similar material and number of holes, our optimized *Q* is two orders of magnitude higher. It is important to note as well that existing literature reporting nanobeam designs are all coming from human’s manually tuned results while we are using deep RL to fulfill this task.

**Analysis of training time and sample efficiency.** With regards to the training time, the optimal trial of L2DO w/DQN was trained for 58 episodes in 88 h (shown in Figure 4(a) and Table 2) to inverse design the L3 nanobeam. On the other hand, the optimal trial of L2DO-DSA w/PPO was trained for 131 episodes in 152 h (also shown in Figure 4(b) and Table 2) to inverse design the long nanobeam. Training time was kept in control thanks to the early stopping technique we used, as pointed out in Figure 4. The total time spent on tuning the model was of course slightly longer because of the multiple trials we did and our limited parallel computing and CPU/GPU capacity. Critically, L2DO-DSA w/PPO utilized the DSA technique to increase sample efficiency and speed up training convergence. Without DSA, the training time is estimated to be over 300 h and the sample efficiency 2.5 times lower for the exact same hyperparameter settings in the same model. Furthermore, compared to the human expert and supervised deep learning, L2DO-DSA is estimated to have achieved over 200 times higher sample efficiency based on our calculations. A higher sample efficiency will play a bigger role when more complex photonic structures [4, 5] are inverse designed or larger state-action spaces are adopted in the future.

**Generated optical responses and their meanings.** Next, when verifying the solved designs in FDTD, we also calculated the resulting Electric field profiles and emission spectra, which are visualized in Figure 5(b) and (c). The corresponding FDTD-verified *Q*, *V*, and *λ* values are also labelled on the plots. According to the spectra in Figure 5(b), a single resonance peak can be located at the target wavelengths (950 and 1550 nm, respectively, for each nanobeam), indicating the existence of a single mode and the correctness of the electromagnetic (EM) modes given by the nanobeams designed by L2DO. *y*-axes of the spectra typically represent the intensity of the EM field measured experimentally or simulated by computer while *x*-axes correspond to wavelengths. For low-*Q* cavities, *Q* is inversely proportional to full width at half maximum (FWHM) of the spectrum. For high-*Q* cavities such as the L3 and long nanobeams, we cannot directly extract *Q* from the emission spectrum because the FWHM of each resonance in the spectrum is limited by the simulation time, *T*_sim_, by FWHM ∼ 1/*T*_sim_. Please see the Supporting Information for how *Q* is related to the spectrum in this case. According to the *E*_*y*_ profiles in Figure 5(c), we see that a fundamental or second order resonance mode is achieved by both nanobeam structures, which means that the design parameters solved by L2DO indeed gave rise to correct resonance mode profiles with respect to the spectra in Figure 5(b). These results guarantee a high-quality and precise laser for applications in interconnects on PICs and telecommunications. In future endeavors, experimentalists in our group will fabricate the solved designs demonstrated here into actual laser devices capable of performing real-world light-emitting tasks.

**Discussion on fabrication uncertainties/tolerances.** To account for potential fabrication uncertainties (such as during the ICP-RIE and EBL processes), the L2DO-retrieved design parameters were randomly perturbed within ± 10% of its neighborhood. Corresponding FDTD-calculated optical responses of the perturbed structures showed that *Q*-factors of about half of the structures stayed above the requested thresholds, thus demonstrating the robustness of the retrieved designs against fabrication uncertainties. For instance, when the solved *x*_1_ ∼ *x*_6_ and *r* of the long nanobeam were randomly fluctuated by ±10% on 20 independent trials, the calculated *Q*-factors of 9 cases remained higher than 5e+7; among the remaining 11 cases that dropped below 5e+7, the lowest and highest *Q*-factors were 3.24e+7 and 4.57e+7, respectively. The percentage error between 4.57e+7 and 5e+7 is calculated to be 8.6%, which is less than 10%. Therefore, it is observed that although perturbing design parameters could lead to degraded *Q*-factors in some of the cases, the highest *Q*-factor is still within the margin of error we expected. This insensitivity to fabrication errors will be crucial for future experimental verification of our inverse designed nanobeam cavities.

**Discussion on the generalizability of L2DO.** To optimize new designs without further training or with minimal training means to expand the generalizability of L2DO. In its current form, L2DO already possesses decent generalizability because if we were to slightly perturb the air hole radii such that it stays within a relatively close neighborhood of the current optimal solution, we could obtain optimized results for the new design by only training for a few iterations. This can be justified by the fact that RL can effectively learn from past cumulative experiences of high diversity in a large domain. As a result, designs with small changes in design parameters have most likely been encountered by the RL agent at some point during training and thus can be quickly identified by L2DO if needed. Nonetheless, in the current stage, we still need to re-train the whole algorithm for each new design. In order to obtain one model which is trained on setting A but can easily be adapted to target setting B or C, the model needs to have a good transfer ability. To the best of our knowledge, there are two paradigms in the field of machine learning that allow one model to achieve this goal, namely (1) the pre-train and fine-tune paradigm and (2) the meta-learning paradigm. In the pre-train and fine-tune paradigm, a model is pre-trained in a high-diversity dataset which contains samples pre-collected from different prior tasks, and then adapted to the target task by fine-tuning a part of the model or some additional parameters using a task-specific objective function. In our inverse-designed cavity problem, we have collected the dataset from previous RL trainings for each cavity setting, and have trained a general agent with those data. When faced with a new task, we can use this agent to perform some online interactions and update the policy to achieve adaptation. As for meta-learning [63], this new paradigm presents a greater potential to learn common information from various tasks and transfer to some previously unseen tasks similar to the pre-train method. However, due to the larger computational requirement, meta-trained models are usually only used for optimizing the same type of device with different design parameters (such as a long vs. a short nanobeam). In addition, we believe it is more advantageous to use the pre-trained method for optimizing across devices that are more differentiated or less similar in kind.

**Comparison with particle swarm optimization (PSO).** PSO is a meta-heuristic optimization algorithm that makes little assumptions about the problem being solved and can quickly search very large spaces of candidate solutions while offering no guarantees that an optimum be found. Basically, PSO trades optimality and accuracy for speed. PSO has been demonstrated in several prior works [64] concerning photonics optimization. We ran our nanobeam nanocavities with PSO and included the optimization results in the Supporting Information. As seen in Figure 1 in the Supporting Information, PSO did not match the level L2DO had reached. Nonetheless, we believe if given the right amount of computing power and time frame and under ideal circumstances, PSO may come close to L2DO’s results. Last but not least, since deep RL has been demonstrating groundbreaking performance [38, 39, 45, 47] in a variety of fields in recent years, we do believe L2DO will play a competitive role in photonics inverse design in the long run.

**Figure 6:**
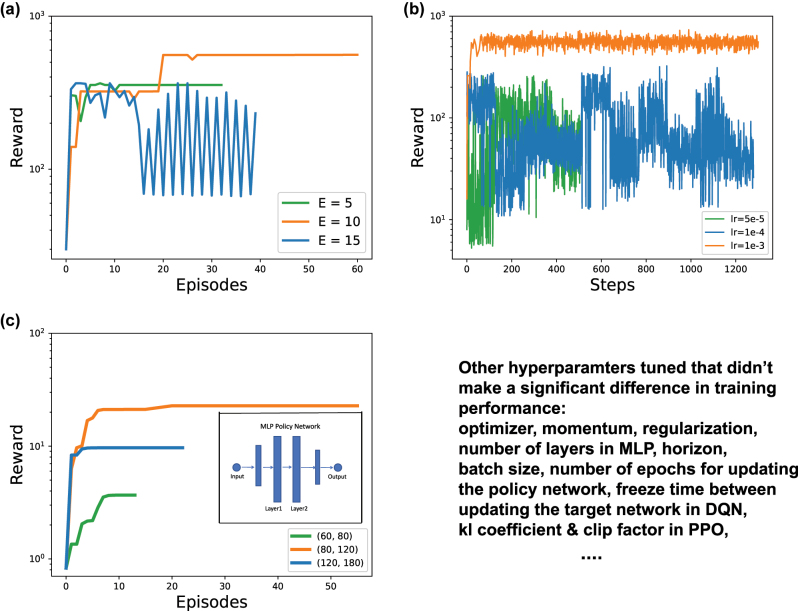
Comparative studies on tuning key hyperparameters in the process of finding the optimal settings for L2DO(-DSA). (a) Learning curves for different *E* values (i.e., 5, 10, and 15) set in the L2DO-DSA framework with PPO. The curve that reached the maximum converged reward in the shortest time possible has the optimal *E* we are looking for. (b) Learning curves for different learning rates *lr* (i.e., 5e−5, 1e−4, and 1e−3) set in L2DO-DSA with PPO for updating the policy network. Rewards versus steps is plotted in this case to more closely examine the effects of different *lr’s*. (c) Learning curves for different numbers of neurons in the middle 2 layers of the MLP policy network set in L2DO with DQN. Three combinations are chosen: (60, 80), (80, 120), and (120, 180). Inset: middle 2 layers of the policy network. Lower right corner lists some additional hyperparameters tuned by us that did not lead to a significant improvement in training.

**Comparative studies on tuning key hyperparameters.** Finally, comparative studies were done on different hyperparameters in order to search for the optimal settings for L2DO(-DSA). Three of the studies are discussed here in detail. First, a comparative study on the impact different *E*’s have on the training of L2DO is presented. See Supporting Information for the definition of *E*. We chose *E* = 5, 10, 15 and ran three experiments with L2DO-DSA (using PPO) to inverse design the long nanobeam. All the other hyperparameters were kept constant throughout these experiments. Learning curves from these three runs are plotted in Figure 6(a) for a comparison of their respective convergences and rewards. Early stopping is utilized to curtail the training time. As seen in Figure 6(a), a maximum reward is achieved in the shortest time when *E* = 10, which indicates we should set *E* as 10 for DSA. *E* = 5 and 15 yielded poorer results by comparison. When *E* is 5, the surrogate DNN (refer to Figure 3) is illy trained and perhaps underfit due to insufficient training data accumulated in the first 5 episodes. When *E* is 15, the surrogate DNN was trained on sufficient data but it took too long till the DNN came into play, which inevitably hurt the training convergence and sample efficiency of the whole learning process. Therefore, *E* = 10 is proven to be the optimal choice for the DSA framework as it found a delicate balance between the amount of training data accumulated and the precise time when the DNN gets activated. This conclusion aligns well with the common dilemma between overfitting and underfitting in machine learning. Second, a comparative study on different learning rates *lr* set in L2DO-DSA with PPO is conducted, where three *lr* = 5e−5, 1e−4, and 1e−3 are chosen. The *lr* is used in updating the policy network with gradient descent. Because lr affects every single step of training, the three runs with different lr’s are plotted in terms of rewards versus steps in Figure 6(b) to better examine the fine details of the learning curves. As seen in Figure 6(b), *lr* = 1e−3 achieved the fastest and smoothest convergence with highest rewards, making itself the optimal lr in this case. The other two *lr*’s, 5e−5 and 1e−4, both displayed very noisy and spiky learning curves and thus are believed to be poor choices. According to further experiments, *lr*’s larger than 1e−3 could cause catastrophic divergence to the training process. The above phenomena could be explained by the fact that smaller *lr*’s can slow down training convergence while larger *lr*’s can cause training to diverge or skip the optimum. Third, a comparative study on different numbers of neurons in the middle two layers of the MLP policy network set in L2DO with DQN is done. Here, three different combinations are chosen: (60, 80), (80, 120), and (120, 180), where the first number in each pair corresponds to Layer1 and the second number Layer2 as shown in the inset in Figure 6(c). Figure 6(c) showcases the learning curves for these three different combinations of neuron quantities and the trial with (80, 120) beat the rest with a clear margin. The (60, 80) trial did poorly likely because it is a somewhat under-parameterized model and thus failed to learn an effective mapping. On the other hand, although the (120, 180) trial had enough parameters to fit the model, it also has a lot more parameters to train and thus made the whole convergence process slower. The (80, 120) trial, however, appears to have just the right amount of parameters the problem needs and was able to rapidly converge to the highest rewards in a short time. We subsequently chose (80, 120) as the network dimension for our policy. These experiments above demonstrate the importance of constantly tuning and finding the optimal hyperparameters when one attempts to train a deep RL model; without properly chosen hyperparameters, the model has a good chance of diverging to undesirable directions very quickly. The lower right corner of Figure 6 lists some additional hyperparameters tuned by us that did not improve the model performance by an appreciable degree; nonetheless, these are still important hyperparameters that one should carefully tune in an RL model.

## 3 Discussion

In summary, this work features the first deep RL model, L2DO, applied to inverse designing and optimizing optical resonators playing a crucial role in nanoscale laser cavities. The inverse problem concerns retrieving a design topology that satisfies certain optical specifications of the photonic device. L2DO successfully addressed three major challenges faced by state-of-the-art deep learning-enabled inverse design methods: (1) a severe shortage of available training data and/or the need for a pre-trained neural network model; (2) the issue of one-to-many mapping or non-unique solutions; (3) the inability to perform optimization of the photonic structure beyond inverse designing. Specifically, L2DO incorporates two advanced RL algorithms, DQN and PPO, to inverse design photonic crystal nanobeam cavities. According to our studies, L2DO not only met the required maxima of certain optical responses (e.g., the *Q* factor > 50 million) but also optimized some good-to-have features (e.g., modal volume and wavelength) that are conducive to a high-quality laser. The solved values were then verified manually in FDTD and their correctness was confirmed by checking the generated optical responses. We can conclude that L2DO successfully improved state-of-the-art results in the literature by over 2 orders of magnitude and dominated a human expert by delivering *Q* factors that exceed the human’s results by over 10 times, while requiring only a fraction of the time and effort. In addition, to tackle issues like low sample efficiency and time-consuming simulations, a novel DNN/Simulator Alternating technique was introduced to L2DO and demonstrated very effective and consistent performance across different scenarios. L2DO-DSA is estimated to have reached over 200 times higher sample efficiency based on our calculations and reduced training time by a substantial amount. In the last few paragraphs, we discussed the connection between our optimized results and fabrication tolerances and compared L2DO to existing optimization algorithms such as the PSO algorithm. In the last part of this paper, we conducted several comparative studies on the effects different hyperparameters have on the training process and demonstrated how we arrived at the optimal network settings for L2DO. We then offered some advice on what hyperparameters need special attention when one attempts to tune a deep RL model. All in all, our inverse designed laser cavities can find broad applications in modern PICs, interconnects, and telecommunications.

Looking forward, L2DO will be expanded and strengthened in the following domains. (1) More advanced versions of deep Q-learning such as DDQN [65], Dueling DQN [66], ApeX DQN [67], and rainbow [68] etc. will succeed the current DQN; (2) The alternating mechanism in DSA (adopting an odd/even approach) will be succeeded by more sophisticated alternating mechanisms. Moreover, the following interesting thrusts will be explored as well. First, we look to incorporate in L2DO a light-weight neural network (NN) model [69] with high interpretability, which features a small model depth, limited parameters, and fast training. This NN can replace the current MLP policy network and the surrogate DNN in DSA. Next, we could introduce Gaussian processes (GP) [70] into the state space of L2DO-DSA that calculates an uncertainty; when the uncertainty goes over a threshold, DSA will switch to the FDTD simulation and when the uncertainty drops, DSA will switch back to the surrogate DNN. This method can provide an attractive alternative to the current odd/even switching mechanism. Third, a new paradigm of RL, coined offline RL [71], has recently emerged as a promising path towards effective real-world RL applications. Offline RL requires learning knowledge solely from previously collected datasets, without any active real-time environment interactions. Offline RL can improve the generalizability and speed of RL models by an appreciable degree. Last, our group will fabricate the inverse designed nanobeams and experimentally verify their validity.

Our methodology in this work is inspired by the famous marriage of AI and Electronic Design Automation (EDA) [38] over the last 5–8 years. For future prospects, this work paves the way for applying deep RL to the rapid multi-objective inverse design and optimization of nanophotonic devices without the need for pre-collecting any data or resorting to human-centered trial-and-error iterations. Through our efforts, we mainly aim to empower the rise of fully automated photonic design because the current state of Photonic Design Automation (PDA) is still largely lacking. Our time and efforts in the subsequent stage will be chiefly directed towards developing software that brings AI and PDA together into one unity.

## Supporting information

The Supporting Information is available at the online version of this article and the repository: https://github.com/Arcadianlee/Photonics-FDTD-DRL.git. Correspondence and requests for materials should be addressed to Z.Z.

## Supplementary Material

Supplementary Material Details

## References

[1] Ma W., Liu Z., Kudyshev Z. A., Boltasseva A., Cai W., Liu Y. (2021). Deep learning for the design of photonic structures. *Nat. Photonics*.

[2] So S., Badloe T., Noh J., Bravo-Abad J., Rho J. (2020). Deep learning enabled inverse design in nanophotonics. *Nanophotonics*.

[3] Noda S. (2006). Seeking the ultimate nanolaser. *Science*.

[4] Hirose K., Liang Y., Kurosaka Y., Watanabe A., Sugiyama T., Noda S. (2014). Watt-class high-power, high-beam-quality photonic-crystal lasers. *Nat. Photonics*.

[5] Yang L., Li G., Gao X., Lu L. (2022). Topological-cavity surface-emitting laser. *Nat. Photonics*.

[6] Dave H., Gao Z., Fryslie S. T. M., Thompson B. J., Choquette K. D. (2019). Static and dynamic properties of coherently-coupled photonic-crystal vertical-cavity surface-emitting laser arrays. *IEEE J. Sel. Top. Quantum Electron.*.

[7] Milzarek A., Ulbrich M. (2014). A semismooth Newton method with multidimensional filter globalization for L1-optimization. *SIAM J. Optim.*.

[8] Jiang J., Fan J. A. (2019). Global optimization of dielectric metasurfaces using a physics-driven neural network. *Nano letters*.

[9] Jiang J., Chen M., Fan J. A. (2021). Deep neural networks for the evaluation and design of photonic devices. *Nat. Rev. Mater.*.

[10] Molesky S., Lin Z., Piggott A. Y., Jin W., Vucković J., Rodriguez A. W. (2018). Inverse design in nanophotonics. *Nat. Photonics*.

[11] Zunger A. (2018). Inverse design in search of materials with target functionalities. *Nat. Rev. Chem*.

[12] Noh J., Kim J., Stein H. S. (2019). Inverse design of solid-state materials via a continuous representation. *Matter*.

[13] Sekar V., Zhang M., Shu C., Khoo B. C. (2019). Inverse design of airfoil using a deep convolutional neural network. *AIAA J.*.

[14] Sapra N. V., Vercruysse D., Su L. (2019). Inverse design and demonstration of broadband grating couplers. *IEEE J. Sel. Top. Quantum Electron.*.

[15] Freeze J. G., Ray Kelly H., Batista V. S. (2019). Search for catalysts by inverse design: artificial intelligence, mountain climbers, and alchemists. *Chem. Rev.*.

[16] Mei J., Wu Y., Chan C. T., Zhang Z.-Q. (2012). First-principles study of Dirac and Dirac-like cones in phononic and photonic crystals. *Phys. Rev. B*.

[17] Xu Y., Peng B., Zhang H., Shao H., Zhang R., Zhu H. (2017). First-principle calculations of optical properties of monolayer arsenene and antimonene allotropes. *Ann. Phys.*.

[18] Zhang Z., Qiu M. (2004). Small-volume waveguide-section high Q microcavities in 2D photonic crystal slabs. *Opt. Express*.

[19] Song B.-S., Noda S., Asano T., Akahane Y. (2005). Ultra-high-Q photonic double-heterostructure nanocavity. *Nat. Mater.*.

[20] Wiecha P. R., Arbouet A., Girard C., Lecestre A., Larrieu G., Paillard V. (2017). Evolutionary multi-objective optimization of colour pixels based on dielectric nanoantennas. *Nat. Nanotechnol.*.

[21] Hegde R. S. (2019). Photonics inverse design: pairing deep neural networks with evolutionary algorithms. *IEEE J. Sel. Top. Quantum Electron.*.

[22] Dobson D. C., Cox S. J. (1999). Maximizing band gaps in two-dimensional photonic crystals. *SIAM J. Appl. Math.*.

[23] Felici T., Engl H. W. (2001). On shape optimization of optical waveguides using inverse problem techniques. *Inverse Probl.*.

[24] Goodfellow I., Bengio Y., Courville A. (2016). *Deep Learning*.

[25] Krizhevsky A., Sutskever I., Hinton G. E. (2012). Imagenet classification with deep convolutional neural networks. *Advances in Neural Information Processing Systems*.

[26] Peurifoy J., Shen Y., Jing L. (2018). Nanophotonic particle simulation and inverse design using artificial neural networks. *Sci. Adv.*.

[27] Liu D., Tan Y., Khoram E., Yu Z. (2018). Training deep neural networks for the inverse design of nanophotonic structures. *ACS Photonics*.

[28] Liu Z., Zhu D., Rodrigues S. P., Lee K.-T., Cai W. (2018). Generative model for the inverse design of metasurfaces. *Nano Lett.*.

[29] Dai P., Sun K., Yan X. (2022). Inverse design of structural color: finding multiple solutions via conditional generative adversarial networks. *Nanophotonics*.

[30] Ma W., Cheng F., Xu Y., Wen Q., Liu Y. (2019). Probabilistic representation and inverse design of metamaterials based on a deep generative model with semi-supervised learning strategy. *Adv. Mater.*.

[31] Ma W., Liu Y. (2020). A data-efficient self-supervised deep learning model for design and characterization of nanophotonic structures. *Sci. China Phys. Mech. Astron.*.

[32] Asano T., Noda S. (2019). Iterative optimization of photonic crystal nanocavity designs by using deep neural networks. *Nanophotonics*.

[33] Sutton R. S., Barto A. G. (2018). *Reinforcement Learning: An Introduction*.

[34] Degrave J., Felici F., Buchli J. (2022). Magnetic control of tokamak plasmas through deep reinforcement learning. *Nature*.

[35] Koneru A., Batra R., Manna S. (2022). Multi-reward reinforcement learning based bond-order potential to study strain-assisted phase transitions in phosphorene. *J. Phys. Chem. Lett.*.

[36] Sommer C., Asjad M., Genes C. (2020). Prospects of reinforcement learning for the simultaneous damping of many mechanical modes. *Sci. Rep.*.

[37] Whitelam S., Tamblyn I. (2020). Learning to grow: control of material self-assembly using evolutionary reinforcement learning. *Phys. Rev. E*.

[38] Mirhoseini A., Goldie A., Yazgan M. (2021). A graph placement methodology for fast chip design. *Nature*.

[39] Mnih V., Kavukcuoglu K., Silver D. (2015). Human-level control through deep reinforcement learning. *Nature*.

[40] Wang H., Yang J., Lee H.-S., Han S. (2018). Learning to design circuits. ..

[41] Do N., Truong D., Nguyen D., Hoai M., Pham C. (2021). Self-controlling photonic-on-chip networks with deep reinforcement learning. *Sci. Rep.*.

[42] Proctor P., Teuscher C., Hecht A., Osiński M. (2021). Proximal policy optimization for radiation source search. *J. Nucl. Eng.*.

[43] Gupta U., Mandal S. K., Mao M., Chakrabarti C., Ogras U. Y. (2019). A deep Q-learning approach for dynamic management of heterogeneous processors. *IEEE Comput. Architect. Lett.*.

[44] Kuprikov E., Kokhanovskiy A., Serebrennikov K., Turitsyn S. (2022). Deep reinforcement learning for self-tuning laser source of dissipative solitons. *Sci. Rep.*.

[45] Sajedian I., Lee H., Rho J. (2020). Design of high transmission color filters for solar cells directed by deep Q-learning. *Sol. Energy*.

[46] Silver D., Schrittwieser J., Simonyan K. (2017). Mastering the game of go without human knowledge. *Nature*.

[47] Vinyals O., Babuschkin I., Czarnecki W. M. (2019). Grandmaster level in StarCraft II using multi-agent reinforcement learning. *Nature*.

[48] Li R., Gu X., Shen Y., Li K., Li Z., Zhang Z. (2022). Smart and rapid design of nanophotonic structures by an adaptive and regularized deep neural network. *Nanomaterials*.

[49] Chen X., Li R., Yu Y. (2022). POViT: vision transformer for multi-objective design and characterization of photonic crystal nanocavities. *Nanomaterials*.

[50] Khatib O., Ren S., Malof J., Padilla W. J. (2022). Learning the physics of all-dielectric metamaterials with deep Lorentz neural networks. *Adv. Opt. Mater.*.

[51] Pilozzi L., Farrelly F. A., Marcucci G., Conti C. (2018). Machine learning inverse problem for topological photonics. *Commun. Phys.*.

[52] Malkiel I., Mrejen M., Nagler A., Arieli U., Wolf L., Suchowski H. (2018). Plasmonic nanostructure design and characterization via deep learning. *Light Sci. Appl.*.

[53] Sajedian I., Badloe T., Rho J. (2019). Optimisation of colour generation from dielectric nanostructures using reinforcement learning. *Opt. Express*.

[54] Sui F., Guo R., Zhang Z., Gu G. X., Lin L. (2021). Deep reinforcement learning for digital materials design. *ACS Mater. Lett.*.

[55] Arulkumaran K., Deisenroth M. P., Brundage M., Bharath A. A. (2017). Deep reinforcement learning: a brief survey. *IEEE Signal Process. Mag.*.

[56] Schulman J., Wolski F., Dhariwal P., Radford A., Klimov O. (2017). Proximal policy optimization algorithms. ..

[57] Fegadolli W. S., Kim S.-H., Postigo P. A., Scherer A. (2013). Hybrid single quantum well InP/Si nanobeam lasers for silicon photonics. *Opt. Lett.*.

[58] Quan Q., Deotare P. B., Loncar M. (2010). Photonic crystal nanobeam cavity strongly coupled to the feeding waveguide. *Appl. Phys. Lett.*.

[59] ..

[60] Kim S., Ahn B.-H., Kim J.-Y., Jeong K.-Y., Kim K. S., Lee Y.-H. (2011). Nanobeam photonic bandedge lasers. *Opt. Express*.

[61] McCutcheon M. W., Loncar M. (2008). Design of a silicon nitride photonic crystal nanocavity with a Quality factor of one million for coupling to a diamond nanocrystal. *Opt. Express*.

[62] Quan Q., Loncar M. (2011). Deterministic design of wavelength scale, ultra-high Q photonic crystal nanobeam cavities. *Opt. Express*.

[63] Finn C., Abbeel P., Levine S. (2017). Model-agnostic meta-learning for fast adaptation of deep networks. *International Conference on Machine Learning. PMLR*.

[64] Darki B. S., Granpayeh N. (2010). Improving the performance of a photonic crystal ring-resonator-based channel drop filter using particle swarm optimization method. *Opt. Commun.*.

[65] Van Hasselt H., Guez A., Silver D. (2016). Deep reinforcement learning with double q-learning. *Proceedings of the AAAI Conference on Artificial Intelligence*.

[66] Wang Z., Schaul T., Hessel M., Hasselt H., Lanctot M., Freitas N. (2016). Dueling network architectures for deep reinforcement learning. *International Conference on Machine Learning. PMLR*.

[67] Horgan D., Quan J., Budden D. (2018). Distributed prioritized experience replay. ..

[68] Hessel M., Modayil J., Van Hasselt H. (2018). Rainbow: combining improvements in deep reinforcement learning. *Thirty-Second AAAI Conference on Artificial Intelligence*.

[69] Zhang T., Yin F., Luo Z.-Q. (2021). Fast generic interaction detection for model interpretability and compression. *International Conference on Learning Representations*.

[70] Zhao Y., Fritsche C., Hendeby G., Yin F., Chen T., Gunnarsson F. (2019). Cramér–rao bounds for filtering based on Gaussian process state-space models. *IEEE Trans. Signal Process.*.

[71] Kumar A., Zhou A., Tucker G., Levine S. (2020). Conservative q-learning for offline reinforcement learning. *Adv. Neural Inf. Process. Syst.*.

