# Supplementary material for: Deep reinforcement learning empowers automated inverse design and optimization of photonic crystals for nanoscale laser cavities
Source: Nanophotonics. 2023 Jan 12;12(2):319–34. doi: 10.1515/nanoph-2022-0692 (PMC11501320; doi:10.1515/nanoph-2022-0692)
Supplement: Supplementary file 1 — Supplementary Material Details [file j_nanoph-2022-0692_suppl_001.pdf]

Supporting Information:

Deep reinforcement learning empowers automated inverse design and optimization of photonic crystals for nanoscale laser cavities

Renjie Li [1, 2, †] **Ceyao Zhang** [3, 4, †] **Wentao Xie** [1] **Yuanhao Gong** [1]

Feilong Ding [1] **Hui Dai** [2] **Zihan Chen** [4] **Feng Yin** [*, 4] **Zhaoyu Zhang** [*, 1, 5]

[1] Shenzhen Key Laboratory of Semiconductor Lasers, School of Science and Engineering, The Chinese University of Hong Kong, Shenzhen

[2] Shenzhen Research Institute of Big Data (SRIBD), Shenzhen, Guangdong, China

[3] Future Network of Intelligence Institute (FNii), The Chinese University of Hong Kong, Shenzhen

[4] School of Science and Engineering, The Chinese University of Hong Kong, Shenzhen

[5] Peng Cheng Laboratory, Shenzhen, Guangdong, China

[†] indicates equal contribution [*] indicates corresponding authors: yinfeng@cuhk.edu.cn, zhangzy@cuhk.edu.cn

Deep RL algorithm overview. Due to the availability of a variety of RL algorithms, L2DO is implemented with two algorithms that are known to be stable and fast: DQN and PPO. Specifically, PPO with actor-critic[1] is chosen. Table 1 lists the comparison between key characteristics of DQN and PPO for clarity. To improve the training speed, the authors made the following choice for a balance between simulation time and sample efficiency. As an off-policy algorithm, DQN is known to be more sample efficient and therefore is used for the more time-consuming L3 nanobeam. PPO, on the other hand, is less sample efficient being on-policy and is naturally chosen for the less time-consuming long nanobeam. Basically, the rationale is that the faster (or slower) algorithm is assigned to compensate the nanobeam with slower (or faster) simulations. In spite of their notable difference in sample efficiency, however, PPO can still surpass DQN in training performance owing to its many other merits and one ought not to judge either algorithm by a single attribute alone. Additionally, the authors would like to justify the choice of DQN over its other variants. Although there exists several variants[2] of DQN such as DDQN, dueling DQN, ApeX DQN and Rainbow that have been shown to possess improved performance for some tasks, they also inevitably involve bigger networks and more hyperparameters and thus are harder to train than DQN. Therefore, only DQN is used in this proof-of-concept work and its more intricate variants will be experimented with for future research.

Table 1: Comparison between Deep-Q Learning (DQN) and Proximal Policy Optimization (PPO) algorithms. TRPO in the bottom row of the right column stands for Trust Region Policy Optimization.

	**DQN**	**PPO**
Model-free?	✓	✓
Policy	Off-policy	On-policy
Type	Value-based	Policy-based
Can use DNN?	✓	✓
Discrete actions?	✓	✓
Continuous actions?	✗	✓
Sample efficient?	✓	✗
Actor-critic?	✗	✓
Replay buffer?	✓	✓
Year invented	2015	2017
Predecessor	Q-learning[3]	TRPO[4]

Preprint. Under review.

Deep Q-learning. A brief overview of the DQN algorithm is provided here. Algorithm 1 outlines the pseudo-code of DQN according to an adapted version from Mnih et. al [5]. The code that realizes L2DO strictly follows Algorithm 1 step-by-step. DQN innovatively incorporates a policy (action-value) network $Q(s, a)$ combined with a target network $Q'(s, a)$ that can greatly enhance the training performance. $Q'(s, a)$ is initialized to be a clone of $Q(s, a)$ with the exact same parameters. In the last step of Algorithm 1, parameter C refers to how many steps do we wait until the target action-value function $Q'(s, a)$ gets updated by $Q(s, a)$, also known as the "freeze time". This freezing mechanism was found particularly helpful for stabilizing the convergence and reducing oscillations of the policy.

Another fundamental element of DQN is the experience replay [5] realized by a replay buffer D. At each step, the agent's transition (s, a, r, s') (known as experiences) is stored in a pre-allocated array D. During updating the policy with SGD, experiences in D are randomly drawn to act as training samples. Using experience replay has the following benefits: first, past experiences are reused in many future gradient updates, which allows for a greater sample efficiency and possibly faster convergence; second, since consecutive samples are usually correlated, they often possess highly similar distributions that can cause the learning to be stuck at local minima. Randomizing these samples can break the data correlation and enable a more diverse data distribution. Using experience replay can also smoothen out learning curves and alleviate oscillations or even divergence in training.

Next, ε-greedy is also a central unit of DQN. Since DQN is off-policy, it directly predicts actions from the greedy policy $a = arg\max_{a'} Q(s, a')$. In principle, ε-greedy dynamically adjusts a balance between exploration and exploitation that allows the agent to explore a more extensive range of the state space. The agent selects and executes actions according to the ε-greedy policy based on $Q(s, a)$, according to Algorithm 1. In practice, the ε-greedy policy obeys the greedy policy with probability 1-ε and selects a random action with probability ε otherwise. In this work, the start and end values of ε are set to be 0.90 and 0.05, respectively.

The objective function (i.e., loss function) of the problem is defined as:

$$L(\theta) = \mathbb{E}[(r + \gamma \max_{a'} Q'(s', a) - Q(s, a))^2] \tag{1}$$

One would optimize the loss $L(\theta)$ in Equation 1 by stochastic gradient descent, as shown in Algorithm 1. In our experiments with DQN, we used the RMSprop optimizer with minibatches of size 32 and learning rate of 0.00025. In Equation 1, r is the reward. Discount factor γ is chosen as 0.99, which is used to estimate the cumulative return defined at some future time point *T*. The cumulative return is defined as a discounted sum of all future rewards:

$$R = \sum_{t}^{T} \gamma^t r_t \tag{2}$$

By optimizing the loss $L(\theta)$ in Equation 1, the cumulative return R in Equation 2 will be maximized which in turn would lead to the optimal action we are looking for.

Algorithm 1: deep Q-learning (DQN)

Initialize replay buffer D to capacity N
Initialize action-value function $Q(s, a)$ with random weights θ
Initialize target action-value function $Q'(s, a)$ with weights θ'
For all states $s \in \mathcal{S}^+$ and actions $a \in \mathcal{A}(s)$
for *episode* **do**
    Initialize s;
    for *step of episode* **do**
        Choose a given s using policy based on $Q(s, a)$ (e.g., ε-greedy);
        Take action a in environment, observe r, s';
        Store transition (s, a, r, s') in D;
        Sample random minibatch of transitions from D;
        Do SGD on $[r + \gamma \max_{a'} Q'(s', a') - Q(s, a)]^2$ wrt. θ;
        $s \leftarrow s'$;
        Every C steps set $Q'(s, a) = Q(s, a)$;
    end for
end for

Proximal Policy Optimization. A brief overview of the PPO algorithm is provided below. Apart from the DQN and its variants which make decisions based on the value functions, policy-based methods [6] learn the policy function directly. The Actor Critic (AC) algorithms [7] combine the policy and value structure together, where the policy (actor) selects actions and the value (critic) criticizes the actions made. These type of methods are on-policy in that the policy is updated by data collected from executing the most recent policy. Since the policy and value are learned simultaneously, there is no guarantee that the AC algorithms can achieve monotonic improvement. TRPO [4] solves this problem through constraining the largest step possible to improve the policy. However TRPO involves a complex conjugate high-order gradient calculation and is hard to implement. PPO [8] converts the constrained optimization in TRPO to unconstrained optimization and incorporates some novel techniques to solve the problem. As an on-policy algorithm, PPO tries to unmask how we can take the longest stride possible on a policy update using the samples currently available, without stepping so far that we inadvertently cause performance deterioration. Algorithm 2 outlines the pseudo-code of PPO according to an adapted version from Schulman et. al [8]. The code that realizes L2DO strictly follows Algorithm 2. PPO updates policies via the following objective function:

$$\theta_{k+1} = arg \max_{\theta} \frac{1}{|D_k T|} \sum_{\tau} \sum_{t=0}^{T} \min(\frac{\pi_\theta}{\pi_{\theta_k}} A^{\pi_{\theta_k}}(s_t, a_t), g(\epsilon, A^{\pi_{\theta_k}}(s_t, a_t))) \tag{3}$$

In Equation 3, T is the total steps to take, $\tau \in D_k$ is a trajectory collected, π_θ denotes the policy, and the part after the min operator corresponds to the loss function L. In this work, the loss function is optimized by a SGD optimizer with minibatch size of 32 and learning rate = 1e-3. The advantage term A is oftern estimated by Generalized Advantage Estimation (GAE) [8]. The g term, which represents the *clipping* operation, is defined as:

$$g(\epsilon, A) = \begin{cases} (1 + \epsilon)A & \text{for } A \geq 0 \\ (1 - \epsilon)A & \text{for } A < 0 \end{cases}$$

Clipping serves to reduce incentives for the policy to move in drastically long strides, and the hyperparameter ϵ controls how far the updated policy should move from the old one while still improving the objective. Clipping plays a crucial role in the current state-of-the-art PPO algorithm.

Algorithm 2: Proximal Policy Optimization (PPO)

Initialize policy parameters θ_0 and value function parameters ϕ_0
for *k = 0, 1, 2...* **do**
  Collect trajectories $D_k = \{\tau\}$ by running policy π_k in the environment;
  Compute rewards-to-go R_t;
  Compute advantage estimates A_t based on current value function V_ϕ;
  Update policy by maximizing the PPO objective via SGD:
  $\theta_{k+1} = arg\max_\theta \frac{1}{|D_k T|} \sum_\tau \sum_{t=0}^{T} \min(\frac{\pi_\theta}{\pi_{\theta_k}} A^{\pi_{\theta_k}}(s_t, a_t), g(\epsilon, A^{\pi_{\theta_k}}(s_t, a_t)))$;
  Fit value function by regression on Mean Squared Error via SGD:
  $\phi_{k+1} = arg\min_\phi \frac{1}{|D_k|T} \sum_\tau \sum_{t=0}^{T} (V_\phi(s_t) - R_t)^2$;
end for

State and reward definitions. Table 2 showcases the state spaces and action spaces of L2DO for long nanobeam and L3 nanobeam. Both nanobeams are set to have 7 states and 14 discrete actions to allow for a reasonably sized parameter space and limit the total training time in this proof-of-concept work. Higher-order state-action spaces can be chosen for more thorough investigations in the future. The state space, which is a subset of design parameters, consists of geometric parameters of the nanobeam cavity including the x-locations and radii of air holes. State is fed into the policy network as its input. The action space is formed by increasing or decreasing each state by a fixed step size of 0.25 nm or 0.5 nm as shown in Table 2. The optimal action, which is predicted by the policy, is then fed into the FDTD environment to update the state and calculate the associated rewards. For example, if the current state $s = 1.0$ and the action is $a = +0.5$, the environment will yield the next state as $s' = 1.0 + 0.5 = 1.5$ and the associated reward as rew'. The output reward and next state are redirected into the policy to begin the next loop and are also simultaneously used to update the policy network. Past states and rewards are normally stored in a replay buffer for reuse down the road and to break sample correlations [5], as shown in Figure 2(a). After many steps of actions, if the value of the current state happens to go over the boundary set by the Min and Max values in Table 2, the current episode will be forced to terminate and a new episode will begin. Full details of L2DO's mechanism can be found in the supplementary material.

Next, Equation 4-6 defines the reward and how it's related to the target optical responses as laid out in the inverse problem:

$$rew_1 = (const - (Q^* - Q) \times 1e\text{-}5) \quad (4)$$

$$rew_2 = \frac{\alpha}{|\lambda^* - \lambda|} + \beta(V^* - V) \quad (5)$$

$$rew_T = \gamma \times rew_1 + \eta \times rew_2 \quad (6)$$

where in rew_1, Q^* is the target maximum to reach, Q is the value of the current Q factor yielded by the FDTD environment, $const$ is a constant value used to negate the reward so that larger Q factors can end up with a larger reward too, and $1e$-5 is a scaling factor to normalize the magnitude of rewards. In rew_2, λ^* and V^* are the target wavelength and modal volume, respectively, and α and β are tunable weights. rew_2 is defined such that $\lambda' s$ closer to λ^* and $V' s$ less than V^* would give rise to higher rewards, which matches the inverse design problem defined earlier. It is noteworthy that rew_1 and rew_2 represent the REQUIRED and good-to-have aspects of the inverse problem, respectively. Finally, Equation 6 defines the total reward rew_T as a weighted sum of rew_1 and rew_2, and for our problem more weights are placed on rew_1 (i.e., $\gamma > \eta$) because it's REQUIRED. Values of all coefficients are chosen to be: $\alpha = 2, \beta = 1, \gamma = 50, \eta = 0.1$ after multiple rounds of tuning with different combinations.

DNN/Simulator Alternating technique. In photonics-centered RL models, the environment is typically represented by an FDTD simulator that solves Maxwell's Equations. As shown in Figure 2(a), L2DO goes through the FDTD simulation environment once for each step it takes, and from Table 1 in the manuscript we know each simulation takes over 2.5 mins of time. At this rate, L2DO's RL agent will never be sufficiently trained within a reasonable time-frame, making the inverse

Table 2: State space and action space of L2DO for the long nanobeam (top) and L3 nanobeam (bottom), respectively. There's 7 states and 14 actions for each nanobeam. x_i's are the x-coordinates of air holes in the Taper region, with x_1 being the innermost hole, x_2 the second from the innermost, etc. x_m refers to the x-coordinates of holes in the Mirror region. r refers to the radius of circular holes, and a_1 (a_2) are the semi-minor (major) axes of elliptical holes. Refer to Figure 1 for a detailed illustration of the above geometric quantities. State is bounded by preset Min and Max limits.

**long nanobeam**		
**State space**	**Min** (nm)	**Max** (nm)
x_1	-10	10
x_2	-10	10
x_3	-10	10
x_4	-10	10
x_5	-10	10
x_6	-10	10
r	-5	5
**Action space**: each state ± 0.25 nm	**Total No. of actions**: 14	**Action type**: discrete

**L3 nanobeam**		
**State space**	**Min** (nm)	**Max** (nm)
x_1	-20	20
x_2	-20	20
x_3	-20	20
x_4	-20	20
a_1	-10	10
a_2	-20	20
x_m	-10	10
**Action space**: each state ± 0.5 nm	**Total No. of actions**: 14	**Action type**: discrete

design intractable or even insignificant. In this work, we devised a novel DNN/Simulator Alternating technique (DSA) (see Figure 3) to address this issue, where a surrogate DNN is introduced to approximate the FDTD simulator to reduce the computational cost and boost sample efficiency. This technique, while suitable for both DQN and PPO, plays a central role in our PPO model because of the low sample efficiency induced by the time-consuming simulations and PPO's inherent on-policy nature. According to Figure 3, DSA is implemented with the following details:

I. Data sequences from the FDTD simulator are stored in the replay buffer for the first *E* episodes, during which the FDTD simulator functions as the environment as usual;

II. At the end of the *Eth* episode, all data stored are first concatenated and then used to train the surrogate DNN whose input is next states and output is reward. The trained DNN can now be activated to approximate the simulator.

III. Starting from the *(E+1)th* episode, odd episodes use the DNN as the environment whereas even ones use the FDTD simulator (thus the "alternating" technique). When the latter is used, data sequences are stored to the replay buffer for continuously updating the DNN in odd episodes.

L2DO equipped with DSA will be referred to as L2DO-DSA in this paper. In I, *E* is a parameter to be determined by our subsequent experiments and is set to be 10 in Figure 3 as an example. *E* here is a measure for how long we should wait before the surrogate DNN gets activated. In II, the input to the surrogate DNN is set as *next states* because $s' = s + a$ and the *state* is already known at each iteration before reaching the environment. Therefore, using *next states* as the input is equivalent to using *actions*. The DNN then outputs the approximate rewards without resorting to the reward functions defined in Equation 4-6. In III, the simulator and the DNN are engineered to operate every other episode in this initial demonstration of DSA's capabilities; in future studies, we will adopt a more sophisticated "alternating" mechanism where it's up to the model to decide when the switching happens. Data samples collected in even episodes from the simulator are stored and used to train the DNN in odd episodes. Based on the above implementations, we will show that L2DO-DSA largely reduces the time needed for training and improves the sample efficiency by circumventing the FDTD solver periodically.

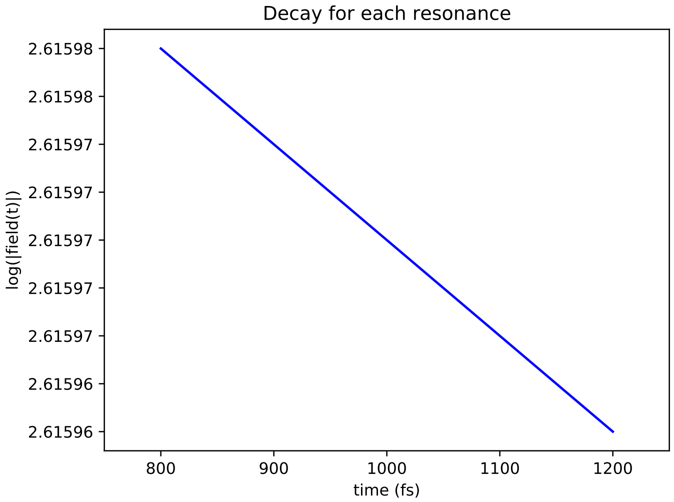

Figure 1: The time decay of the sum of squared field components on a logarithmic scale. The slope of the line (i.e., slope of decay) is used in the Q factor calculation. The high-Q mode (also the fundamental resonance mode) corresponds to the line in the plot.

Relationship between Q factor and the spectrum. While for low-Q cavities the Q factor can be determined from the FWHM by the relationship $Q = f/\text{FWHM}$ where f is the resonance frequency, for high-Q cavities, we cannot directly extract Q from the emission spectrum because the FWHM of the spectrum is limited by the simulation time, T_{sim}, by FWHM $\sim 1/T_{sim}$. In this case, the Q factor is calculated by the following formula:

$$Q = \frac{-2\pi f log(e)}{2m} \tag{7}$$

where m is the slope of the log of the time signal envelope (i.e., the slope of decay). In FDTD, we did the following to calculate the slope of decay: first calculate the envelope of the time-domain field signal. Then isolate each resonance peak in the frequency domain using a Gaussian filter, and then take the inverse Fourier transform to calculate the time decay separately for each peak. The plot of the slope of decay is included in Figure 1. The blue line corresponds to the high Q mode of 5e+7 of our long nanobeam, which yields a negative slope close to zero in terms of 1/fs (on the order of -1e-8 1/fs or -1e+7 1/s). The curve in Figure 1 is linear because it's potted on a log scale, and in reality the decay is actually exponential.

Choice of laser cavities for inverse design. Scanning Electron Microscopy (SEM) images of a sample long nanobeam and a sample L3 nanobeam fabricated by our group are displayed in Extended Data Figure 1(a)-(b), where the scale bar is given in terms of a unit micron. Extended Data Figure 1(c) illustrates how nanobeam laser cavities–while coupled to waveguides–can be applied to Photonics Integrated Circuits (PICs)[9] under a "Silicon-on-Insulator"[10, 11] design paradigm. PICs are widely used in next-gen mega-sized data centers and advanced telecommunications. Nanobeam cavities are commonly used as laser sources in PICs, interconnects, and telecommunications when coupled to waveguides and optic fibers. Shown is a simplified schematic of a PIC.

Computing resources and software packages used. The RL code was fully written in Python strictly following the algorithmic model illustrated in Figure 2-3, and popular machine learning libraries like Pytorch, OpenAI gym, and Ray RLlib were extensively utilized in our program. In particular, gym and RLlib greatly expedited the code developing process. The training of L2DO was performed on 2 Dell workstations with 8 Intel Xeon Gold 5222 cores and an NVIDIA Quadro P4000 GPU. Further computations for the comparative studies were done on a cluster machine with 30 Intel Xeon Gold 5218 processors and 6 NVIDIA 2080Ti graphics cards. The cluster machine is about 200% faster than the Dell workstations.

As for the FDTD simulations, FDTD (Finite Difference Time Domain) is a method which uses the central difference quotient to replace the first-order partial derivative of the field to time and space,

and obtains the field distribution by recursively simulating the wave propagation process in the time domain. This way, the wave propagation process can be analyzed easier and faster.

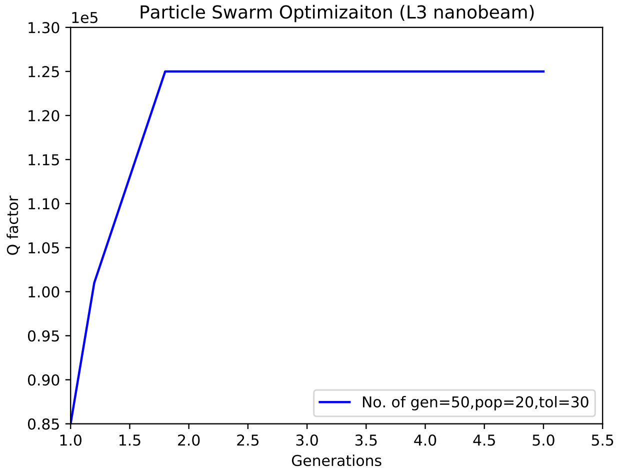

Figure 2: PSO optimization results on the L3 nanobeam, where a highest Q of 1.25e+5 was obtained. This is smaller than the Q of 3e+6 achieved by L2DO. Max number of generations is 50, population size is 20, and tolerance is 30.

Comparison with Particle Swarm Optimization (PSO). Optimization results obtained for the L3 nanobeam using PSO are shown here. As seen in Figure 2 above, after running the PSO for about 40 hours, a highest Q of 1.25e+5 was obtained at around the second generation and remained unchanged afterwards. This is smaller than the Q of 3e+6 achieved by L2DO. We obverse that although PSO was able to reach a maximum relatively quickly, it was unable to further explore the state space to search for the optimal solution. RL, on the other hand, has an artificial agent and a DNN policy that are able to explore and optimize to a fuller extent over a longer period of time.

Comparison with iterative optimizations with DNNs. Asano et al. reported an iterative optimization and inverse design scheme for PC nanocavities using Convolutional Neural Networks (CNN) [12]. In their work, the author prepares an initial dataset, trains the CNN to learn a mapping between design parameters and Q, searches for structures that increase the Q factor by changing design parameters via gradient descent, verifies the Q's by simulations, and updates the dataset by including the new structures with higher Q's. The author was able to increase the Q from 7000 to 11 million using this approach with over 100 iterations. Our present work has several key advantages over this approach. First, L2DO doesn't require any initial dataset to start training. Second, searching for the optimal action via a value-based or policy-based RL agent is to a certain extent more efficient and smarter than doing gradient descent in every step. Third, L2DO was sufficiently trained on MLP networks, which are proven to be faster to run than CNNs. Finally, verifying the design in the FDTD environment and updating the dataset are automatically taken care of by L2DO, whereas in the cited work[12] the authors would have to do it manually. Therefore, we can interpret L2DO as a more streamlined, light-weight, and intelligent version.

Comparison with tandem MLP networks. Liu et al. demonstrated a tandem DNN structure for the inverse design of nanophotonic structures (dielectric thin films specifically) [13]. Figure 1(c) in the main text illustrates this DNN model. More specifically, the authors showed that by combining a pre-trained forward network and an inverse network in a tandem fashion, one can overcome the fundamental issue of non-uniqueness, allowing DNNs to effectively inverse design complex photonic structures. The authors argued that their model solves the issue of non-uniqueness because the designs by the DNN are not required to be the same as the ground truths in the training set. Instead, the loss function would be low as long as the generated design and the ground truth have similar responses. Our present work has several key advantages over this approach. First, this cited work [13] used a large-scale dataset of 550,000 samples for training the DNN, which would take weeks or even months to generate for complex structures. Second, the cited work required having a pre-trained DNN, which will be prohibitive to obtain if there's no dataset existing. Last but not least, the cited

work demonstrated results of inverse designing but failed to discuss their model's ability to optimize the photonic structure. By contrast, L2DO can optimize the photonic structure's Q factor and modal volume on top of simply inverse designing the structure.

Comparison with Generative Adversarial Networks and Variational Autoencoder. GANs and VAEs are both deep generative models widely applied in image generation. They have recently been adopted by the photonics community to inverse design a variety of structures (particularly metasurfaces) [14–17]. The core idea behind generative models is to sample a distribution from a latent space and generate various new "designs" from it. Figure 1(c) in the main text illustrates this approach. However, besides needing a large training dataset (> 15,000 samples) that could be hard to obtain, these models are unable to perform optimization of the photonic structures beyond inverse designing. For instance, Ma et al. used a VAE to inverse design metamaterials that met the target reflectance spectra [16]; similarly, Dai et al. used GAN to inverse design color filters that met the target colors [15]. In both works, the inverse designed structure simply mimics or replicates the initial structure it started with and the optical properties/features of the structure do not receive any improvement or optimization. This can be interpreted as follows: generative models like VAE and GAN aim to produce a variety of designs, which sacrifice accuracy for diversity; if only optimized for a fixed goal, they might not be the best option. L2DO, on the other hand, can inverse design and optimize structures at the same time.

Extended Data Table 1 | List of PPO hyperparameters and their values.

Hyperparameter	Value
discount factor γ	0.99
bootstrapping param	0.95
clip ratio	0.2
vf clip ratio	10.0
lambda	1.0
kl coeff	0.2
kl target	0.01
vf loss coeff	1.0
entropy coeff	0.0
policy MLP dims	(60,80,120,80)
policy optimizer	SGD
policy learning rate	1e-3
minibatch size	32
num of epochs	10
episode length	250
num of episodes	500
DSA optimizer	SGD
DSA learning rate	1e-3
DSA MLP dims	(80,60,40)
DSA E param	10
DSA minibatch size	32
num of epochs	20

Extended Data Table 2 | List of DQN hyperparameters and their values.

Hyperparameter	Value
discount factor γ	0.99
ϵ start, end	0.90, 0.05
ϵ decay	400
policy MLP dims	(80,120,80)
policy optimizer	RMSprop
learning rate	0.00025
momentum	0.9
alpha	0.95
minibatch size	32
episode length	150
num of episodes	500
replay buffer size	1500
target update freq	150
train freq	20

The values of most of the hyperparameters, except those shown in Figure 6, were selected by performing an informal search with the nanobeams. We did not perform a systematic or exhaustive grid search owing to the high computational cost, although it is understandable that even better results could be obtained by systematically tuning the hyperparameter values one-by-one.

References

[1] Tuomas Haarnoja et al. "Soft actor-critic: Off-policy maximum entropy deep reinforcement learning with a stochastic actor". In: *International conference on machine learning*. PMLR. 2018, pp. 1861–1870.

[2] Matteo Hessel et al. "Rainbow: Combining improvements in deep reinforcement learning". In: *Thirty-second AAAI conference on artificial intelligence*. 2018.

[3] Christopher JCH Watkins and Peter Dayan. "Q-learning". In: *Machine learning* 8.3 (1992), pp. 279–292.

[4] John Schulman et al. "Trust region policy optimization". In: *International conference on machine learning*. PMLR. 2015, pp. 1889–1897.

[5] Volodymyr Mnih et al. "Human-level control through deep reinforcement learning". In: *nature* 518.7540 (2015), pp. 529–533.

[6] Richard S Sutton and Andrew G Barto. *Reinforcement learning: An introduction*. MIT press, 2018.

[7] Vijay Konda and John Tsitsiklis. "Actor-critic algorithms". In: *Advances in neural information processing systems* 12 (1999).

[8] John Schulman et al. "Proximal policy optimization algorithms". In: *arXiv preprint arXiv:1707.06347* (2017).

[9] Tin Komljenovic et al. "Heterogeneous silicon photonic integrated circuits". In: *Journal of Lightwave Technology* 34.1 (2016), pp. 20–35.

[10] Simon M Sze, Yiming Li, and Kwok K Ng. *Physics of semiconductor devices*. John wiley & sons, 2021.

[11] George K Celler and Sorin Cristoloveanu. "Frontiers of silicon-on-insulator". In: *Journal of Applied Physics* 93.9 (2003), pp. 4955–4978.

[12] Takashi Asano and Susumu Noda. "Iterative optimization of photonic crystal nanocavity designs by using deep neural networks". In: *Nanophotonics* 8.12 (2019), pp. 2243–2256.

[13] Dianjing Liu et al. "Training deep neural networks for the inverse design of nanophotonic structures". In: *Acs Photonics* 5.4 (2018), pp. 1365–1369.

[14] Zhaocheng Liu et al. "Generative model for the inverse design of metasurfaces". In: *Nano letters* 18.10 (2018), pp. 6570–6576.

[15] Peng Dai et al. "Inverse design of structural color: finding multiple solutions via conditional generative adversarial networks". In: *Nanophotonics* (2022).

[16] Wei Ma et al. "Probabilistic representation and inverse design of metamaterials based on a deep generative model with semi-supervised learning strategy". In: *Advanced Materials* 31.35 (2019), p. 1901111.

[17] Wei Ma and Yongmin Liu. "A data-efficient self-supervised deep learning model for design and characterization of nanophotonic structures". In: *Science China Physics, Mechanics & Astronomy* 63.8 (2020), pp. 1–8.
